# Leaf Photosynthetic Rate of Tropical Ferns Is Evolutionarily Linked to Water Transport Capacity

**DOI:** 10.1371/journal.pone.0084682

**Published:** 2014-01-09

**Authors:** Shi-Bao Zhang, Mei Sun, Kun-Fang Cao, Hong Hu, Jiao-Lin Zhang

**Affiliations:** 1 Key Laboratory of Economic Plants and Biotechnology, Kunming Institute of Botany, Chinese Academy of Sciences, Kunming, Yunnan, China; 2 Key Laboratory of Tropical Forest Ecology, Xishuangbanna Tropical Botanical Garden, Chinese Academy of Sciences, Mengla, Yunnan, China; 3 University of Chinese Academy of Sciences, Beijing, China; University College Dublin, Ireland

## Abstract

Ferns usually have relatively lower photosynthetic potential than angiosperms. However, it is unclear whether low photosynthetic potential of ferns is linked to leaf water supply. We hypothesized that there is an evolutionary association of leaf water transport capacity with photosynthesis and stomatal density in ferns. In the present study, a series of functional traits relating to leaf anatomy, hydraulics and physiology were assessed in 19 terrestrial and 11 epiphytic ferns in a common garden, and analyzed by a comparative phylogenetics method. Compared with epiphytic ferns, terrestrial ferns had higher vein density (D_vein_), stomatal density (SD), stomatal conductance (g_s_), and photosynthetic capacity (A_max_), but lower values for lower epidermal thickness (LET) and leaf thickness (LT). Across species, all traits varied significantly, but only stomatal length (SL) showed strong phylogenetic conservatism. A_max_ was positively correlated with D_vein_ and g_s_ with and without phylogenetic corrections. SD correlated positively with A_max_, D_vein_ and g_s_, with the correlation between SD and D_vein_ being significant after phylogenetic correction. Leaf water content showed significant correlations with LET, LT, and mesophyll thickness. Our results provide evidence that A_max_ of the studied ferns is linked to leaf water transport capacity, and there was an evolutionary association between water supply and demand in ferns. These findings add new insights into the evolutionary correlations among traits involving carbon and water economy in ferns.

## Introduction

Ferns are an important component of the forest flora, having critical functions in ecosystem processes, especially in tropical rainforests [1]. Their remarkable degree of diversity and abundance reflect their ecological success in both the past and present [1], [2]. However, ferns are mostly prominent in humid and shade habitats with low evaporative potential [1], [3], and inherently have slower growth rates and lower photosynthetic potentials than angiosperms [2], [4], [5]. Although the ecological strategy and niche of a species are relevant to its physiology and functional traits, our understanding of fern physiology is still fragmentary [6], and the primary determinant of photosynthetic potential in fern is not fully understood [2].

Plant hydraulics can impose fundamental constraints on the photosynthetic gas exchange, growth and distribution of land plants [7]–[9], and ferns have lower leaf hydraulic conductance to liquid water than angiosperms [4], [10]. The geographical distribution of ferns is significantly related to the relative water content at which stomata close, leaf thickness, stomatal density and size in a Mexican cloud forest [11]. The reasons in part for the preference of humid environments by ferns would be poorly controlled evaporative potential, low water-use efficiency and xylem hydraulic limitation [2], [12], [13]. However, it is unclear whether low photosynthetic potential of ferns is linked to their leaf hydraulics.

Leaf hydraulic conductance is highly dependent on the anatomy of the leaf [14]. For instance, leaf venation system plays a key role in transporting water to the site of evaporation. Leaf vein traits provide a basis for variation in leaf hydraulic conductance, gas exchange rate and plant performance across species or in the contrasting environments [7], [14], [15]. Previous studies have suggested that minor vein density (D_vein_, vein length per unit leaf area) is a critical factor determining hydraulic conductance, and therefore water supply of a leaf [8]–[10]. Higher D_vein_ can correspond to a higher water supply capacity since it can increase the surface area for exchange of xylem water with surrounding mesophyll, reducing the distance through which water travels outside the xylem [9], [16]. As water supply to evaporative surfaces is essential to maintain stomatal opening, D_vein_ often shows a positive correlation with maximum stomatal conductance and maximum photosynthetic rate (A_max_) across species [7], [8], [17]. Historically, the evolution of D_vein_ results in high A_max_ during the diversification of early angiosperms [10], [18]. However, Walls (2011) found that the relationship between D_vein_ and A_max_ in angiosperms is marginally nonsignificant with phylogenetic regression at a global scale [19]. Compared with angiosperms, ferns have a relatively primitive vascular system composed of tracheid-based xylem, fixed amount of vascular issue, heavily pitted lateral walls bearing pit membranes, and lower D_vein_ [5], [13], [20]–[22]. These features would give ferns higher resistances to water flow, lower water transport capacity and stomatal conductance [4], [5]. Therefore, low water transport capacity may be one of the possible reasons that ferns have low A_max_ values [3]. However, to our knowledge, no study has tested the correlation between photosynthetic rate and vein density in ferns within an evolutionary context.

Both leaf vein architecture and hydraulic conductance can respond rapidly to environmental factors such as light, temperature, humidity or nutrient supply [9], [14], [16], [23]. For example, previous studies have shown that hydraulic adjustment of fronds is a key component in how ferns adapt to contrasting light environments [24]. Hawaiian *Plantago* taxa in drier regions have higher D_vein_ values [25], and the D_vein_ in *Paphiopedilum* tends to increase from terrestrial to epiphytic habitats [26]. At a global scale, D_vein_ correlated negatively with mean annual precipitation and species' shade tolerance index [9]. Consequently, plasticity in vein traits may reflect the optimal solutions to achieving balance between vein investment and environmental demand, and the adaptation of a species to environments in different habitats [9], [27], [28].

Most of the water in plants is diffused through stomata, so stomata play a critical role in maintaining a well-balanced hydration status of the leaf. Stomatal density and size dictate primarily maximum stomatal conductance, and therefore potential transpirational demand [28]–[30]. Increased stomatal density enhances photosynthetic rate by modulating gas diffusion [30]–[32]. Generally, leaves built for higher rates of gas exchange may have smaller stomata [33]. In seed plants, smaller stomata can react more quickly to environmental stimuli, and enable the leaf to attain high diffusive conductivity under favorable conditions, while larger stomata close slowly, and are less able to prevent hydraulic dysfunction in dry habitats [29], [33], [34]. However, several papers have showed that ferns can close stomata in response to dehydration much faster than angiosperms [35], but likely can not close stomata completely. Ferns also have small leaf water potential margin between stomatal closure and leaf death due to water stress. This is because fern stomata are predominantly regulated by a passive response to leaf water status, while angiosperm stomata are actively mediated by abscisic acid [35], [36].

The water status of a leaf is dependent on both stomatal regulation and water supply from the vasculature to inner tissues [14]. The relationship between the density of vein and stomata can reflect an efficient balance between investment in liquid and vapour conductances in the leaf [23], [37]. Selection for high rates of photosynthetic gas exchange of a species may cause a shift in a number of traits which contribute to high leaf hydraulic conductance, because increasing only one should lead to a great limitation by other traits [9], [25]. When the maximum evaporative capacity of the leaf is greater than the capacity of the vascular system to maintain leaf hydration, the stomata will close [23], [38]. Previous studies have found that D_vein_ is correlated with stomatal density [7], [17]. Ferns can close their stomata to reduce water loss, and prevent xylem cavitation and associated dysfunction much earlier than can the stomata of angiosperms [38]. Up to date, no study has shown how stomatal traits are correlated with vein density and photosynthetic gas exchange in ferns.

Leaf structural traits such as mesophyll thickness and epidermal characteristics can affect leaf hydraulic resistance and gas exchange [16], [39]–[41]. For example, leaf hydraulic resistance is related to palisade mesophyll thickness and the ratio of palisade to spongy mesophyll thickness [7]. Thicker leaves are able to store more water and maintain more stable hydraulic functioning during drought periods [39], [42]. In ferns such as *Pyrrosia*, a water-storing tissue is described to include large parenchymal cells [3]. Species with thick leaves usually have large stomata [34], while leaf thickness is negatively correlated with SD [26]. These facts imply that leaf structural traits are linked to the water supply and storage of the leaves. However, the correlation between leaf structure and the maintenance of water balance remains largely unclear in ferns.

In the present study, we used a comparative phylogenetics method to investigate 16 leaf traits of 30 tropical ferns consisting of 19 terrestrial and 11 epiphytic species in a common garden. Our objectives were to examine the correlated evolution between stomatal density and vein density, and to assess the effects of water transport capacity on photosynthesis of tropical ferns. We tested the following hypotheses: (1) vein density is positively correlated with photosynthetic rate because of the strong influence of vein density on leaf hydraulic conductance and stomatal conductance; (2) vein density is positively correlated with stomatal density, reflecting a balance between water supply and transpirational demand.

## Materials and Methods

### Ethics statement

All materials in the present study were collected from Xishuangbanna Tropical Botanical Garden (XTBG), and none of the experimental materials was collected from national parks or other protected areas. The uses of experimental materials were permitted for scientific research by both XTBG and Xishuangbanna National Nature Reserve. No species under first-class state protection were used in this research, and they were not listed in the Inventory of Rare and Endangered Plants of China, or the Key Protected Inventory of Wild Plants of China.

### Plant materials

We gathered samples of 30 fern species, including 19 terrestrial and 11 epiphytic ferns, from 13 families. The names and their ecological characteristics are presented in Table S1 in File S1. This collection was made in a seasonal tropical rainforest at the Xishuangbanna Tropical Botanical Garden (21°41′N, 101°25′E, elevation 570 m) in southern Yunnan Province, China. All species grow under the canopy of the forest, and can receive about 10% of full sunlight. The mean annual temperature is 21.7°C, and the mean annual precipitation is 1560 mm, with 80% falling during the rainy season (May to October). The fronds were collected from at least six individuals per species. All sampling and measurements were conducted from June to August in 2011.

### Leaf physiology

Measurements of leaf physiology were performed on the same individuals used for our anatomical assessments. A Li-Cor 6400 portable photosynthesis system attached with a 6400-40 fluorescence chamber (Li-Cor Inc., Lincoln, NE, USA) was used to measure maximum photosynthetic rate (A_max_), stomatal conductance (g_s_), and transpiration rate (T_r_) on 6 mature leaves from different individuals of each species. All measurements were conducted from 09:30 to 11:30 am, when CO_2_ uptake was maximal and water availability was not limited. Before measurements, each leaf was exposed to a light intensity of 300 µmol m^−2^ s^−1^ for 30 min to induce the maximum stomatal opening. This light level was confirmed as the saturation point for photosynthesis of ferns in the preliminary experiments. During the measurement period, the CO_2_ concentration in the chamber was set to 400 µmol mol^−1^, with leaf temperature at 25 to 27°C, light intensity at 300 µmol m^−2^ s^−1^, flow rate at 200 mol s^−1^ and leaf-to-air vapor pressure deficit at 0.7 to 1.0 kPa.

Leaf water content (LWC) is determined on 6 mature leaves per species from different plants. These samples were collected in the morning, and immediately determined fresh weight, and then oven-dried at 70°C for 48 h to obtain dry weight. We calculated LWC as (fresh weigh-dry weight)/fresh weight ×100.

### Leaf anatomy and morphology

Six mature, undamaged leaves were collected from individual plants of each species. Each leaf was divided along the midrib. Area of one half was measured with a Li-Cor 3000A area meter (Li-Cor Inc., Lincoln, NE, USA), oven-dried at 70°C for 48 h to obtain its dry mass, and calculated its leaf mass per unit area (LMA). Another half was cleaned for 1 h in a 5% NaOH aqueous solution. Three sections of leaf lamina were excised from the top, middle, and bottom portions, stained with 1% safranin, and mounted in glycerol to obtain the vein density (D_vein_). Samples were photographed at 10× magnification using a Leica DM2500 microscope (Leica Microsystems Vertrieb GmbH, Wetzlar, Germany). Vein lengths were determined from digital images via the IMAGEJ program (http://rsb.info.nih.gov/ij/). Values for D_vein_ were expressed as vein length per unit area.

The adaxial and abaxial epidermises were peeled from the middle portions of fresh, mature leaves, and images were made under the Leica DM2500 microscope. For each species, 6 leaves from different individuals were used for stomatal observations. Their stomata were tallied in 30 randomly selected fields. Stomatal density (SD) was calculated as the number per unit leaf area. Stomatal length (SL) was represented by the guard cell length, possibly indicating the maximum potential opening of the pore [43].

From samples of each species, the middle portions of mature leaves were fixed in FAA (formalin, acetic acid, alcohol, and distilled water, 10∶5∶50∶35, v∶v∶v∶v) for at least 24 h. They were then dehydrated in an ethanol series and embedded in paraffin for sectioning. Transverse sections, made on a Leica RM2126RT rotary microtome (Leica Inc., Bensheim, Germany), were mounted on glass slides. These tissues were examined and photographed using the Leica DM2500 microscope. Thicknesses of the cuticle (CT), upper epidermis (UET), lower epidermis (LET), mesophylls (MT), and the whole-leaf (LT) were measured at the midpoint of each transverse section with the IMAGEJ program. Six leaves per species were taken from different individuals. Leaf density (LD) was calculated as LMA/LT.

### Data analysis

A phylogenetic tree for these 30 fern species was constructed based on chloroplast rbcL sequences obtained from the GenBank website (http://www.ncbi.nlm.nih.gov/genbank/). Phylogenetic analyses for each matrix were carried out using the maximum likelihood method in PAUP* v.4.0b10 [44]. Schneider et al. (2004) has integrated *Colysis* and major components of *Microsorum* into *Leptochilus* by using nucleotide sequences derived from three plastid loci [45]. For simplicity, the old Latin names of species in *Colysis* and *Microsorum* were used in the present study.

All statistical analyses were performed with R software v. 2.15.0 [46]. The phylogenetic signal (*K*-statistic) for each trait was calculated using ‘picante’ based on the R package. Such *K*-statistics can express the conservatism of traits. Cases where the *K-*value is <1 indicate convergent traits while *K*>1 represents that traits are more conserved than would be presumed from a Brownian expectation [47].

Relationships among variables were evaluated by both pair-wise Pearson correlations in the R package and a phylogenetically independent contrast (PIC). Possible evolutionary associations were assessed via PIC analysis, utilizing the molecular phylogenetic tree. This PIC analysis was examined with the “analysis of traits” module in Phylocom, which calculates the internal node values for continuous traits [48].

## Results

Leaf functional traits varied considerably across species (Table 1, Tables S2 and S3 in File S1). The magnitude of variation was generally smaller for physiological traits than that of the structural traits. Among species, variation ranges of 15 traits were less than 10.0-fold, while that for SD differed by 15.5-fold. When including morphology and anatomy, the variation in CT was smallest while that of SD was largest. For physiology, g_s_ had the largest variation (9.5-fold), and LWC was the smallest (1.4-fold). In sum, the variation was greatest for SD and smallest for LWC across all traits.

**Table 1:** Leaf traits examined in this study.

Trait	Code	Unit	Mean (minimum-maximum)
Leaf area	LA	cm^−2^	133.01 (25.07–286.17)
Leaf mass per unit area	LMA	g m^−2^	39.27 (21.22–83.17)
Cuticle thickness	CT	µm	1.46 (1.03–2.12)
Leaf density	LD	kg m^−3^	184.17 (62.18–348.47)
Upper epidermal thickness	UET	µm	24.21 (12.54–35.54)
Lower epidermal thickness	LET	µm	18.97 (8.78–51.85)
Leaf thickness	LT	µm	255.33(99.85–585.16)
Mesophyll thickness	MT	µm	200.48 (56.24–516.71)
Stomatal density	SD	no. mm^−2^	65.96 (11.69–180.99)
Stomatal length	SL	µm	42.95 (25.11–63.84)
Vein density	D_vein_	mm mm^−2^	1.12 (0.66–1.68)
Leaf water content	LWC	%	78.82 (65.04–91.04)
Area-based maximum photosynthetic rate	A_max_	µmol m^−2^ s^−1^	3.05 (1.78–5.53)
Mass-based maximum photosynthetic rate	A_mass_	nmol g^−1^ s^−1^	88.27(25.68–151.04)
Stomatal conductance	g_s_	mmol m^−2^ s^−1^	74.97 (16.82–159.30)
Transpiration rate	T_r_	mmol m^−2^ s^−1^	0.82 (0.35–2.05)

Of the 16 leaf traits tested here, significant differences among 11 were found between terrestrial and epiphytic ferns (Table 2). Compared with epiphytic ferns, terrestrial species tended to have higher values for D_vein_, SD, g_s_, A_max_, and T_r_, but lower values for LMA, LET, LT, MT, and SL. However, values for leaf area, LD, UET, LWC and CT did not differ significantly between the two types of ferns.

**Table 2:** Differences in 16 leaf traits between terrestrial and epiphytic ferns.

Trait	Terrestrial	Epiphytic	*p*
Leaf area	144.46±13.92	113.23±21.20	ns
Leaf mass per unit area	33.66±1.88	48.95±4.27	**
Leaf density	205.34±16.62	147.61±16.85	ns
Leaf water content	78.01±1.39	79.95±1.82	ns
Cuticle thickness	1.48±0.07	1.44±0.08	ns
Upper epidermal thickness	23.99±1.54	24.58±1.82	ns
Lower epidermal thickness	16.92±2.12	22.51±1.66	*
Leaf thickness	187.74±20.75	372.06±47.99	***
Mesophyll thickness	138.22±19.31	308.04±46.80	**
Stomatal density	87.20±12.55	29.28±3.07	**
Stomatal length	38.69±1.93	50.32±1.99	**
Area-based maximum photosynthetic rate	3.42±0.19	2.41±0.21	**
Mass-based maximum photosynthetic rate	107.26±5.78	55.47±8.22	***
Stomatal conductance	88.90±98.19	50.91±4.99	***
Transpiration rate	0.95±0.08	0.60±0.06	**
Leaf vein density	1.19±0.06	0.99±0.05	*

See Table 1 for trait units. The statistical differences for each trait were determined with independent-samples *t*-test. The sign of the significance is indicated as: ns, *p*>0.05; *, *p*<0.05; **, *p*<0.01; ***, *p*<0.001.

**Figure 1:**
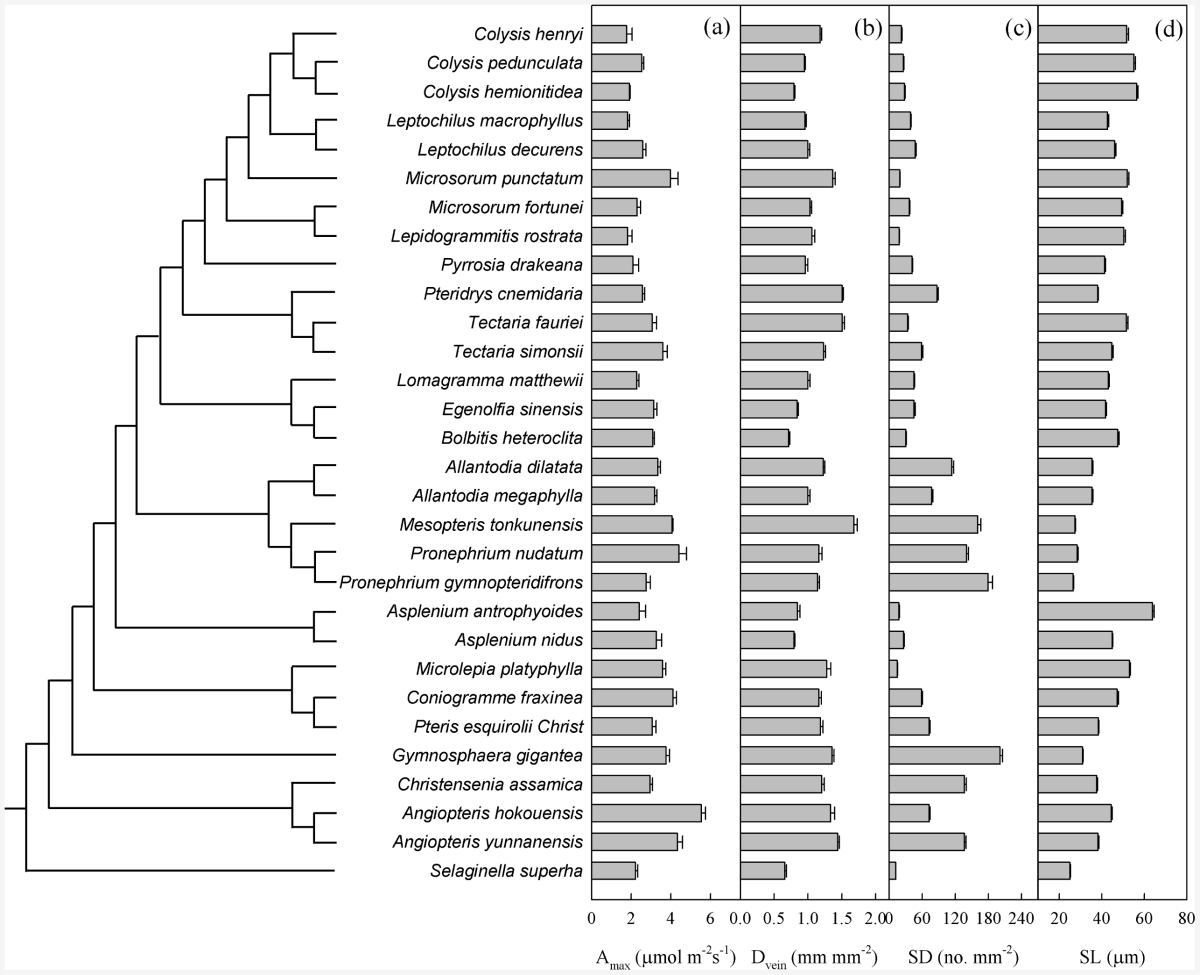
Phylogeny with labeled nodes used for comparative analysis of trait variation among 30 fern species along with trait values (mean ± 1 SE) for maximum photosynthetic rate (A_max_; a), vein density (D_vein_; b), stomatal density (SD; c), and stomatal length (SL; d).

Among all tested traits, only the *K* value for SL was >1.0, demonstrating that this traits were phylogenetically conserved (Figure 1, Table 3). For the others, values were <1.0, indicating that they were convergent.

**Table 3:** Phylogenetic signals (*K*-statistics) for 14 leaf functional traits from 30 fern species.

Trait	*K*-statistic	*p*
Leaf mass per unit area	0.396	0.009
Leaf density	0.289	0.045
Leaf water content	0.292	0.065
Cuticle thickness	0.329	0.422
Upper epidermal thickness	0.425	0.008
Lower epidermal thickness	0.282	0.134
Leaf thickness	0.382	0.016
Mesophyll thickness	0.363	0.019
Stomatal density	0.454	0.004
Stomatal length	1.322	0.001
Area-based maximum photosynthetic rate	0.360	0.012
Stomatal conductance	0.660	0.010
Transpiration rate	0.296	0.032
Vein density	0.632	0.005

*K* value <1 indicates that relatives resemble each other less than expected under Brownian motion evolution along the phylogenetic tree; *K* value >1 shows that close relatives are more similar than expected.

**Figure 2:**
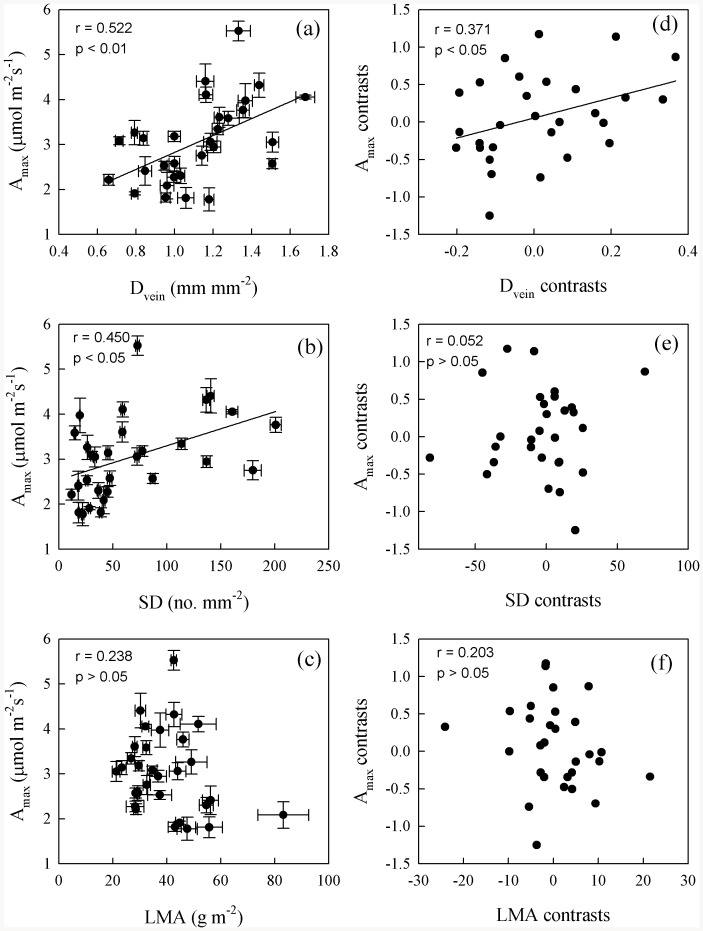
Pearson correlations (a–c) and phylogenetically independent contrast correlations (d–f) of maximum photosynthetic rate (A_max_) with vein density (D_vein_), stomatal density (SD), and leaf mass per unit area (LMA) across 30 fern species.

**Figure 3:**
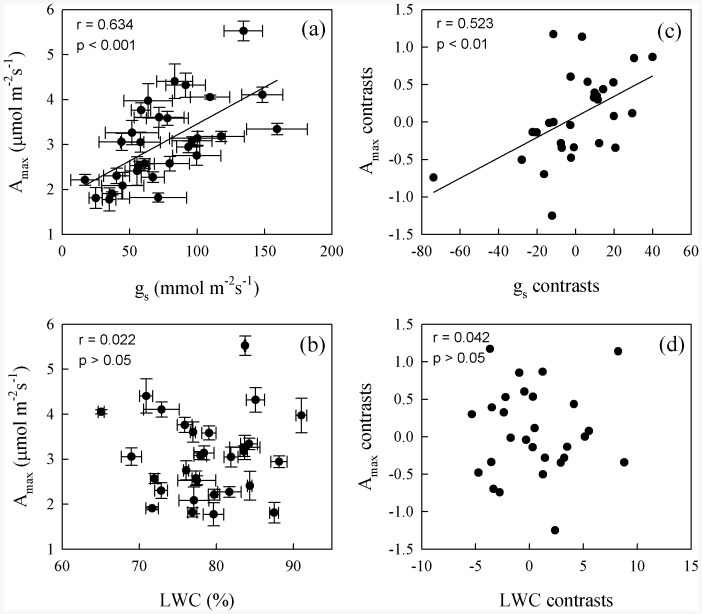
Pearson correlations (a–b) and phylogenetically independent contrast correlations (c–d) of maximum photosynthetic rate (A_max_) with stomatal conductance (g_s_) and leaf water content (LWC) across 30 fern species.

**Figure 4:**
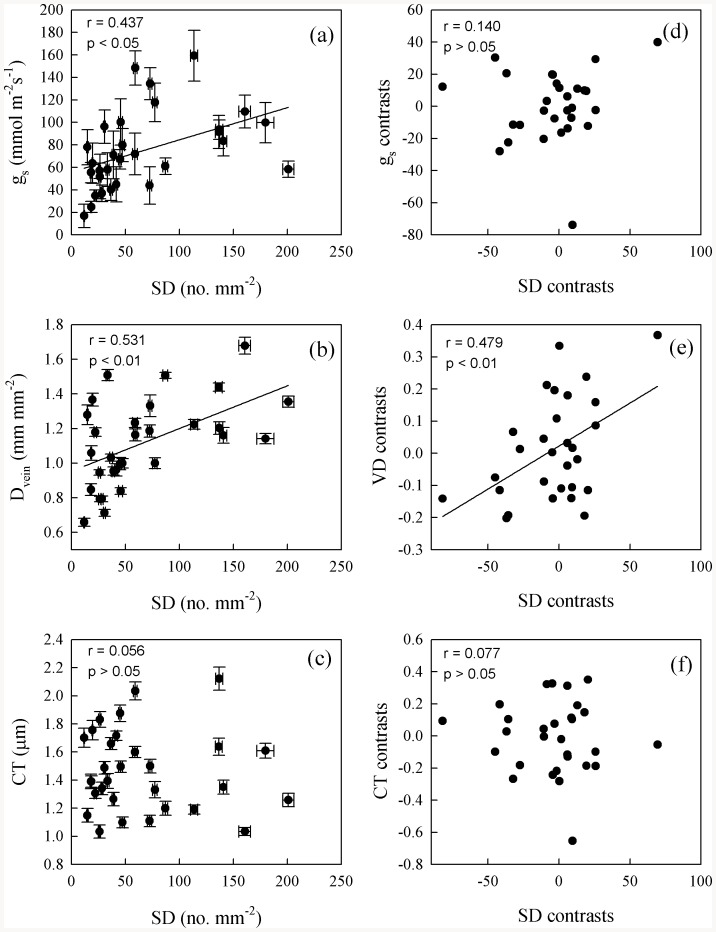
Pearson correlations (a–c) and phylogenetically independent contrast correlations (d–f) of stomatal density (SD) with stomatal conductance (g_s_), vein density (D_vein_) and cuticle thickness (CT) across 30 fern species.

**Figure 5:**
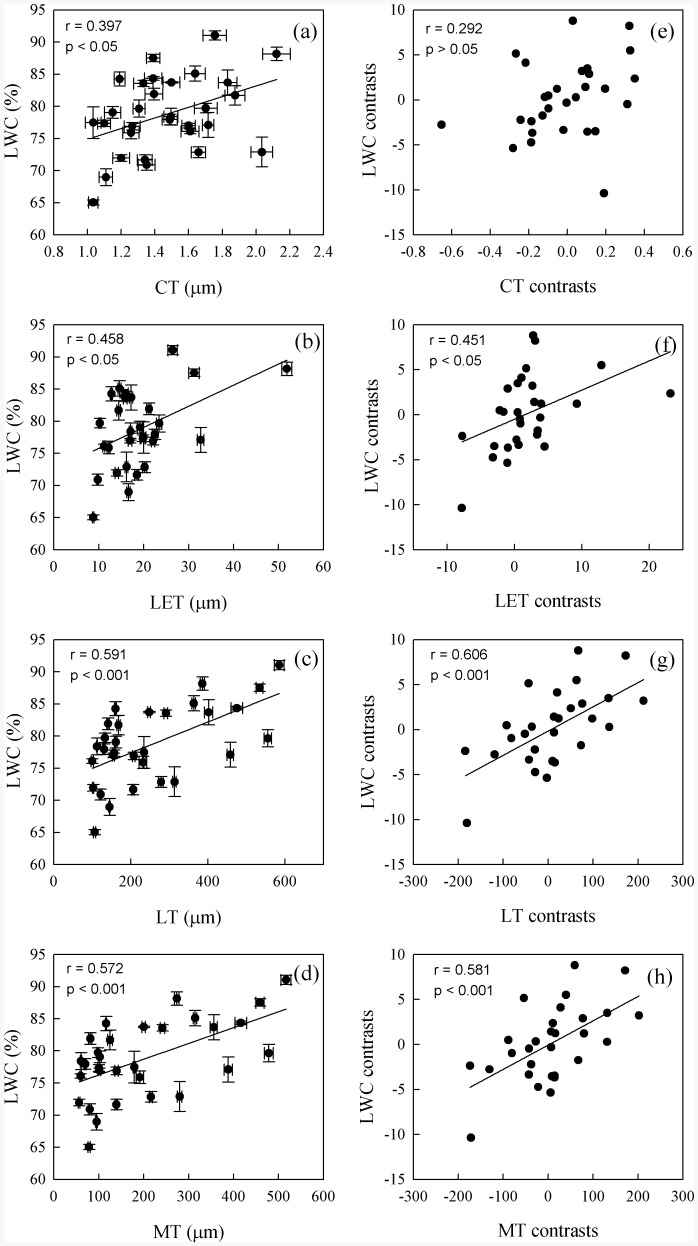
Pearson correlations (a–d) and phylogenetically independent contrast correlations (e–h) of leaf water content (LWC) with cuticle thickness (CT), lower epidermal thickness (LET), leaf thickness (LT), and mesophyll thickness (MT) across 30 fern species.

Maximum photosynthetic rate was positively correlated with D_vein_, SD, and g_s_, but not with LWC and leaf structural traits (Figures 2 and 3, Table S4 in File S1). After phylogeny was considered, A_max_ was still correlated with D_vein_ and g_s_ (Figures 2 and 3). Stomatal density was positively correlated with D_vein_ and g_s_, but not with other structural traits (Figure 4, Table S4 in File S1). After the phylogenetic effects were eliminated, the correlation of D_vein_ with SD was still significant. Phenotypically and phylogenetically, LWC was positively correlated with LET, LT, and MT (Figure 5).

## Discussion

We used a comparative phylogenetics approach to examine the correlated evolution among leaf traits across a range of ferns in a common garden. We found that vein density relating to water transport capacity showed evolutionary associations with maximum photosynthetic rate and stomatal density in tropical ferns.

### Variations in leaf traits between growth habits

Differences in growth habits can reveal variations in the availability of abiotic resources. Generally, water availability is one of the main factors that limit photosynthesis and growth of epiphytic plants [49]. Compared with terrestrial fern, epiphytic species has more resistive vascular systems, higher drought tolerance, and different anatomical features [13]. In this study, epiphytic ferns had higher values for LMA, thicknesses of whole lamina, epidermis and mesophylls than terrestrial species (Table 2). Thick leaves would be favorable in dry habitats because they can store more water [29], [42]. In addition, D_vein_ was lower for the epiphytic type, consistent with the pattern that epiphytic orchids have less venation than their terrestrial counterparts [26]. Torre et al. (2003) suggested that rose grown at high relative humidity (RH) has a significantly higher SD and SL, but a reduced D_vein_ and thinner leaves when compared to moderate RH plant [50]. Contrary to our results, D_vein_ values are higher for Hawaiian *Plantago* taxa on drier sites [25]. Since D_vein_ strongly determines water transport capacity [8], [9], epiphytic ferns have distinctly lower leaf hydraulic conductance due to low D_vein_ than terrestrial ferns. Given that there is a tradeoff between hydraulic capacity and safety [22], epiphytic ferns may have a vascular system that is more resistant to cavitation than terrestrial species [5]. A distinct difference in D_vein_ and consequent water transport capacity is probably responsible for the significant difference in A_max_ between terrestrial and epiphytic ferns. These results reflect an obvious differentiation between epiphytic and terrestrial ferns in ecological adaptations to the environmental conditions of their native habitats.

### Leaf traits in relation to phylogeny

Among leaf traits examined, only stomatal length (SL) showed a strong phylogenetic conservatism (Table 3). This result is consistent with the notion that SL is related to phylogeny in angiosperms [34]. Previous studies have suggested that SL in *Arabidopsis* is strongly correlated with genome size, but is independent from environment [51], and that the frequency of polyploidy in ferns (31%) is much higher than angiosperms (15%) [52]. Polyploidy provides a rapid route for species evolution and adaptation [53]. Thus, speciation linking to polyploidy might explain evolutionary shifts associated with genome size and SL in ferns.

Phylogenetic signals for most of the traits examined here were weak, possibly because of a departure from Brownian motion evolution, such as adaptive evolution, that would not have been correlated with phylogeny. This reflects the outcome of selection in heterogeneous environments, allowing species to acclimate to their current growing conditions [54].

### Correlation of photosynthesis with water supply

As expected, A_max_ was positively correlated with D_vein_, SD, and g_s_, consistent with our hypothesis. Previous studies have suggested that D_vein_ is correlated with maximum hydraulic conductance and A_max_ across a wide range of species [7], [16], [17]. Generally, ferns have lower A_max_ than angiosperms, which are attributable to their much lower D_vein_ and hydraulic conductance [5], [10], [38]. In contrast, angiosperms have dramatically higher values for D_vein_ that parallel their higher rates of photosynthesis and transpiration [4], [16]. Feild & Brodribb (2013) found that high vein density evolution is strongly associated with simplification of the perforation plates of primary xylem vessels. Such simple perforation plates associated with high D_vein_ only occurred in the leaf xylem of derived angiosperm clades, while scalariform perforation plates associated with low D_vein_ occurred in extant basal angiosperms and ferns [55]. Compared with that of the derived angiosperms (>12 mm mm^−2^) [55], the 30 tropical ferns in our study exhibited very lower D_vein_ (0.66–1.68 mm mm^−2^). Thus, due to the lower water supply capacities than angiosperms, ferns cannot efficiently replace the water transpired, which consequently results in a high water potential gradient from roots to leaf and prevents the ferns from achieving and maintaining a high leaf water potential, stomatal conductance, and photosynthetic rate during transpiration [7]. This confirmed the hypothesis in angiosperms that vein density evolution enable higher photosynthesis [10], and low stomatal conductance and photosynthesis of ferns could be caused by low vein density.

### Correlations among leaf functional traits

Our present results support the hypothesis that stomatal density is closely related to D_vein_. We also found that D_vein_ in *Paphiopedilum* (Orchidaceae) is evolutionarily correlated with SD [26]. The close correlation between D_vein_ and SD in ferns and *Paphiopedilum* support the idea of coordinated development and functioning between leaf veins and stomata [17], which is important for optimizing the photosynthetic yield relative to carbon investment in leaf venation, conserving water loss and maintaining xylem function [6]. However, environments would modify the linkage between D_vein_ and SD in woody angiosperms [23]. The most efficient balance of vein and stomatal investment occurs when the supply of water to evaporative sites is just enough to maintain stomata fully open in the contrasting environments [23], [37].

Leaf structural traits can affect photosynthesis through changing the diffusion path from stomata to chloroplast or hydraulic resistance [41]. However, our study did not find any significant correlations between A_max_ and leaf structural traits such as mesophyll thickness (Table S4 in File S1). Leaf water content was positively correlated with thicknesses of the cuticle, upper epidermis, lower epidermis, mesophylls, and the whole-leaf (Figure 5). This demonstrates that leaf structural traits contribute to water conservation. Both leaf thickness and epidermal characteristics affect water status [40]. A thick leaf can store more water and maintain more stable hydraulic functioning during drought periods [42].

### Conclusions

Leaf functional traits of 30 tropical ferns examined varied considerably, but only stomatal length was strongly phylogenetically conserved. We note correlated evolution between maximum photosynthetic rate and vein density, and between stomatal density and vein density in ferns. These results indicate that lower water transport capacity limits the photosynthesis of these tropical ferns. These findings provide novel insights into the correlated evolution of traits involving water economy in early vascular plants such as ferns.

## Supporting Information

File S1 **Combined supporting information file containing Tables S1–S4.** Table S1. A list of species in the present study and their growth forms and native habitat features. Table S2. Species means for leaf morphological traits of 30 ferns. Table S3. Species means for stomatal and physiological traits of 30 ferns. Table S4. Pairwise cross-species and PIC correlations between leaf traits across ferns studied.
(DOC)
