# Supplementary material for: Leaf Photosynthetic Rate of Tropical Ferns Is Evolutionarily Linked to Water Transport Capacity
Source: PLoS One. 2014 Jan 9;9(1):e84682. doi: 10.1371/journal.pone.0084682 (PMC3886989; doi:10.1371/journal.pone.0084682)
Supplement: File S1 — Combined supporting information file containing Tables S1–S4. Table S1. A list of species in the present study and their growth forms and native habitat features. Table S2. Species means for leaf morphological traits of 30 ferns. Table S3. Species means for stomatal and physiological traits of 30 ferns. Table S4. Pairwise cross-species and PIC correlations between leaf traits across ferns studied. (DOC) [file pone.0084682.s001.doc]

**Supporting Information**

***Table S1. A list of species in the present study and their growth form and native habitat features.***

| Species | Family | Growth form | Endemic habitat |
| --- | --- | --- | --- |
| *Colysis henryi* | Polypodiaceae | epiphytic | Tree trunks in forests or rocks of forest understories |
| *Colysis pedunculata* | Polypodiaceae | epiphytic | Rocks along streambank in forests |
| *Colysis hemionitidea* | Polypodiaceae | epiphytic | Rocks along streamside in forests |
| *Leptochilus macrophyllus* | Polypodiaceae | epiphytic | Rocks of moist forest understories |
| *Leptochilus decurens* | Polypodiaceae | epiphytic | Tree trunks in tropical evergreen broad-leaved forests |
| *Microsorum punctatum* | Polypodiaceae | epiphytic | Tree trunks in seasonally dry to moist evergreen forests |
| *Microsorum fortunei* | Polypodiaceae | epiphytic | Rocks along streamside or tree trunks in forests |
| *Lepidogrammitis rostrata* | Polypodiaceae | epiphytic | Tree trunks or rocks in forests |
| *Pyrrosia drakeana* | Polypodiaceae | epiphytic | Tree trunks or rocks in mixed broad-leaved forests |
| *Pteridrys cnemidaria* | Tectariaceae | terrestrial | In dense forests of valley |
| *Tectaria fauriei* | Tectariaceae | terrestrial | In tropical valley rain-forests |
| *Tectaria simonsii* | Tectariaceae | terrestrial | In dense forests of valley or river |
| *Lomagramma matthewii* | Lomariopsidaceae | terrestrial | In dense forests of valley |
| *Egenolfia sinensis* | Dryopteridaceae | terrestrial | In dense forests |
| *Bolbitis heteroclita* | Dryopteridaceae | terrestrial | In dense forests |
| *Allantodia dilatata* | Athyriaceae | terrestrial | In moist broad-leaved forests |
| *Allantodia megaphylla* | Athyriaceae | terrestrial | By streams in forests of valley |
| *Mesopteris tonkunensis* | Thelypteridaceae | terrestrial | In karst forests |
| *Pronephrium nudatum* | Thelypteridaceae | terrestrial | In sparse forests |
| *Pronephrium gymnopteridifrons* | Thelypteridaceae | terrestrial | In dense forests of valley |
| *Asplenium antrophyoides* | Aspleniaceae | epiphytic | Rocks in karst forests |
| *Asplenium nidus* | Aspleniaceae | epiphytic | Tree trunks or rocks in tropical rain forests |
| *Microlepia platyphylla* | Dennstaedtiaceae | terrestrial | In evergreen broad-leaved forests |
| *Coniogramme fraxinea* | Pteridaceae | terrestrial | In evergreen broad-leaved forests |
| *Pteris esquirolii* | Pteridaceae | terrestrial | In dense forests |
| *Gymnosphaera gigantea* | Cyatheaceae | terrestrial | In evergreen broad-leaved forests |
| *Christensenia assamica* | Marattiaceae | terrestrial | In tropical rain forests |
| *Angiopteris hokouensis* | Marattiaceae | terrestrial | In tropical rain forests |
| *Angiopteris yunnanensis* | Marattiaceae | terrestrial | In tropical rain forests |
| *Selaginella superha* | Selaginellaceae | terrestrial | In forests or by streams |

**Table S2. Species means (mean ± 1SE) for leaf morphological traits of 30 fern species.**

| Species | LMA | LD | CT | UET | LET | LT | MT |
| --- | --- | --- | --- | --- | --- | --- | --- |
| *Colysis henryi* | 47.58±3.79 | 88.73±6.22 | 1.31±0.04 | 24.72±0.48 | 23.47±0.58 | 555.63±9.26 | 478.49±9.83 |
| *Colysis pedunculata* | 37.36±4.49 | 162.33±19.81 | 1.03±0.05 | 28.28±0.56 | 19.98±0.52 | 234.39±4.37 | 178.90±2.49 |
| *Colysis hemionitidea* | 44.76±1.45 | 215.12±9.45 | 1.34±0.04 | 26.24±0.45 | 18.50±0.45 | 205.74±3.46 | 139.42±2.99 |
| *Leptochilus macrophyllus* | 43.00±2.46 | 222.67±11.78 | 1.26±0.05 | 34.56±0.64 | 22.10±0.37 | 207.55±3.44 | 139.53±3.65 |
| *Leptochilus decurens* | 29.33±0.57 | 193.77±5.07 | 1.10±0.04 | 31.00±0.50 | 19.79±0.55 | 156.25±3.01 | 100.12±2.64 |
| *Microsorum punctatum* | 37.54±3.27 | 62.18±5.51 | 1.76±0.07 | 23.57±0.53 | 26.45±1.03 | 585.16±12.60 | 516.71±11.27 |
| *Microsorum fortunei* | 54.68±2.60 | 190.45±9.18 | 1.66±0.04 | 19.33±0.37 | 20.21±0.48 | 278.41±6.37 | 216.02±3.64 |
| *Lepidogrammitis rostrata* | 55.70±4.97 | 99.85±8.55 | 1.39±0.04 | 28.04±1.21 | 31.23±1.17 | 534.12±8.72 | 458.84±9.02 |
| *Pyrrosia drakeana* | 83.17±9.42 | 172.77±19.13 | 1.72±0.03 | 23.60±0.64 | 32.68±0.70 | 458.20±8.69 | 388.18±9.21 |
| *Pteridrys cnemidaria* | 28.38±0.49 | 279.62±5.12 | 1.20±0.05 | 23.02±0.61 | 14.04±0.38 | 102.27±1.59 | 56.24±1.69 |
| *Tectaria fauriei* | 21.21±1.44 | 158.47±12.86 | 1.39±0.05 | 34.03±0.61 | 21.20±0.48 | 140.88±3.17 | 81.08±3.80 |
| *Tectaria simonsii* | 28.08±0.84 | 180.48±9.64 | 1.60±0.04 | 33.42±0.56 | 16.98±0.40 | 154.90±2.75 | 101.30±3.12 |
| *Lomagramma matthewii* | 28.29±3.27 | 149.98±15.18 | 1.88±0.06 | 22.32±0.67 | 14.44±0.41 | 168.00±4.64 | 125.19±5.37 |
| *Egenolfia sinensis* | 23.24±1.76 | 215.25±14.75 | 1.50±0.04 | 28.72±0.40 | 17.06±0.32 | 112.07±1.43 | 60.53±1.46 |
| *Bolbitis heteroclita* | 34.60±1.77 | 272.97±19.24 | 1.49±0.04 | 34.27±0.76 | 22.50±0.59 | 130.58±2.85 | 69.73±2.62 |
| *Allantodia dilatata* | 26.75±1.31 | 159.58±8.15 | 1.19±0.03 | 22.25±0.35 | 12.82±0.27 | 160.09±2.64 | 116.49±1.06 |
| *Allantodia megaphylla* | 29.58±1.18 | 100.52±6.83 | 1.33±0.06 | 28.78±0.78 | 15.91±0.41 | 291.10±4.89 | 241.64±5.11 |
| *Mesopteris tonkunensis* | 32.09±1.31 | 281.82±14.18 | 1.03±0.03 | 12.54±0.31 | 8.78±0.18 | 105.87±3.01 | 78.48±2.67 |
| *Pronephrium nudatum* | 30.22±1.94 | 259.23±12.44 | 1.35±0.05 | 15.78±0.44 | 9.77±0.20 | 121.38±3.81 | 80.10±3.29 |
| *Pronephrium gymnopteridifrons* | 32.58±1.35 | 348.47±26.79 | 1.61±0.05 | 19.53±0.28 | 11.15±0.21 | 99.85±3.15 | 59.64±1.67 |
| *Asplenium antrophyoides* | 56.20±2.98 | 104.28±4.04 | 1.39±0.05 | 15.92±0.47 | 16.05±0.49 | 475.10±15.40 | 416.14±13.05 |
| *Asplenium nidus* | 49.11±5.85 | 111.55±13.22 | 1.83±0.06 | 15.15±0.47 | 17.20±0.38 | 402.13±10.86 | 356.07±7.88 |
| *Microlepia platyphylla* | 32.35±1.28 | 204.77±7.72 | 1.15±0.05 | 24.01±0.49 | 19.17±0.58 | 160.78±3.41 | 102.30±3.60 |
| *Coniogramme fraxinea* | 51.67±6.68 | 169.15±20.96 | 2.03±0.06 | 20.72±0.52 | 16.19±0.33 | 313.61±4.42 | 280.03±4.92 |
| *Pteris esquirolii Christ* | 44.02±3.08 | 317.28±25.49 | 1.11±0.04 | 22.03±0.41 | 16.63±0.38 | 144.82±1.98 | 95.18±1.96 |
| *Gymnosphaera gigantea* | 46.01±2.23 | 205.73±17.31 | 1.26±0.05 | 15.81±0.43 | 12.24±0.36 | 231.50±10.32 | 191.45±8.25 |
| *Christensenia assamica* | 36.78±3.14 | 93.10±7.69 | 2.12±0.08 | 35.54±0.99 | 51.85±1.14 | 385.59±5.71 | 273.72±4.91 |
| *Angiopteris hokouensis* | 42.59±1.05 | 174.78±5.64 | 1.50±0.05 | 23.50±0.45 | 15.88±0.40 | 247.75±3.76 | 200.27±3.95 |
| *Angiopteris yunnanensis* | 42.64±2.97 | 112.67±8.53 | 1.64±0.06 | 20.11±0.51 | 14.62±0.37 | 363.45±5.09 | 314.46±3.75 |
| *Selaginella superha* | 28.50±0.84 | 217.60±4.67 | 1.70±0.07 | 19.57±0.43 | 10.24±0.25 | 132.64±2.69 | 98.29±2.27 |

See Table 1 for trait units. LMA, leaf mass per unit area; LD, leaf density; CT, cuticle thickness; UET, upper epidermal thickness; LET, lower epidermal thickness; LT, leaf thickness; MT, mesophyll thickness.

**Table S3. Species means (mean ± 1SE) for stomatal and physiological traits of 30 fern species.**

| Species | D_vein_ | SD | SL | LWC | A_max_ | A_mass_ | g_s_ | T_r_ |
| --- | --- | --- | --- | --- | --- | --- | --- | --- |
| *Colysis henryi* | 1.18±0.02 | 22.01±1.14 | 51.67±0.88 | 79.64±1.34 | 1.78±0.26 | 35.75±3.45 | 34.82±5.05 | 0.35±0.02 |
| *Colysis pedunculata* | 0.95±0.02 | 25.87±1.16 | 55.07±0.74 | 77.46±2.46 | 2.53±0.10 | 74.39±12.45 | 56.95±6.83 | 0.73±0.04 |
| *Colysis hemionitidea* | 0.79±0.02 | 28.20±1.10 | 56.44±0.66 | 71.65±0.81 | 1.91±0.03 | 42.88±1.27 | 36.88±7.30 | 0.51±0.04 |
| *Leptochilus macrophyllus* | 0.95±0.02 | 38.78±1.18 | 42.67±0.52 | 76.89±0.59 | 1.82±0.10 | 42.79±2.89 | 71.07±21.20 | 1.02±0.11 |
| *Leptochilus decurens* | 1.00±0.03 | 47.18±2.12 | 46.00±0.63 | 77.34±0.49 | 2.58±0.16 | 88.2±6.52 | 79.74±14.70 | 0.80±0.04 |
| *Microsorum punctatum* | 1.37±0.04 | 19.44±0.39 | 52.04±0.69 | 91.04±0.72 | 3.97±0.38 | 110.89±15.38 | 63.63±18.00 | 0.64±0.07 |
| *Microsorum fortunei* | 1.03±0.02 | 36.60±1.32 | 49.35±0.51 | 72.84±0.84 | 2.30±0.17 | 37.11±3.41 | 40.23±9.63 | 0.51±0.03 |
| *Lepidogrammitis rostrata* | 1.06±0.04 | 18.25±0.57 | 50.31±0.75 | 87.51±0.58 | 1.81±0.23 | 32.93 ±3.97 | 24.78±4.94 | 0.39±0.03 |
| *Pyrrosia drakeana* | 0.96±0.04 | 41.47±1.35 | 41.35±0.37 | 77.09±1.94 | 2.08±0.30 | 25.68±3.63 | 44.78±15.60 | 0.48±0.07 |
| *Pteridrys cnemidaria* | 1.51±0.02 | 86.96±2.36 | 38.08±0.35 | 71.95±0.51 | 2.57±0.11 | 90.95±5.04 | 61.03±7.32 | 0.73±0.03 |
| *Tectaria fauriei* | 1.51±0.03 | 33.44±1.42 | 51.58±0.74 | 81.91±0.91 | 3.05±0.22 | 145.01±8.39 | 57.95±14.90 | 0.74±0.07 |
| *Tectaria simonsii* | 1.23±0.03 | 58.82±2.15 | 44.72±0.62 | 76.98±0.34 | 3.61±0.22 | 128.45±7.24 | 71.86±18.60 | 0.81±0.06 |
| *Lomagramma matthewii* | 1.00±0.04 | 44.92±1.30 | 43.15±0.42 | 81.69±1.52 | 2.27±0.12 | 86.03±10.17 | 67.20±8.60 | 0.81±0.04 |
| *Egenolfia sinensis* | 0.84±0.02 | 45.49±1.78 | 41.82±0.41 | 78.39±1.31 | 3.14±0.16 | 138.41±10.81 | 100.25±20.60 | 0.85±0.05 |
| *Bolbitis heteroclita* | 0.71±0.02 | 30.53±1.00 | 47.55±0.51 | 77.94±0.81 | 3.08±0.09 | 90.77±7.14 | 96.23±14.80 | 1.00±0.05 |
| *Allantodia dilatata* | 1.22±0.03 | 113.61±3.28 | 35.40±0.43 | 84.25±1.10 | 3.34±0.13 | 130.89±8.49 | 159.30±22.60 | 1.32±0.17 |
| *Allantodia megaphylla* | 1.00±0.03 | 77.22±2.00 | 35.45±0.43 | 83.57±0.49 | 3.18±0.12 | 108.88±8.14 | 117.79±17.20 | 0.95±0.03 |
| *Mesopteris tonkunensis* | 1.68±0.05 | 160.58±5.44 | 27.38±0.39 | 65.04±0.40 | 4.05±0.05 | 127.46±5.65 | 109.63±14.50 | 1.04±0.04 |
| *Pronephrium nudatum* | 1.16±0.05 | 140.69±3.31 | 28.40±0.55 | 70.90±0.86 | 4.41±0.38 | 151.04±20.56 | 83.33±13.30 | 0.94±0.08 |
| *Pronephrium gymnopteridifrons* | 1.14±0.03 | 179.62±7.94 | 26.48±0.29 | 76.09±0.34 | 2.75±0.21 | 85.72±8.67 | 99.80±17.90 | 0.85±0.05 |
| *Asplenium antrophyoides* | 0.85±0.03 | 17.94±0.89 | 63.84±0.80 | 84.35±0.29 | 2.41±0.32 | 44.65±7.80 | 55.38±8.98 | 0.67±0.03 |
| *Asplenium nidus* | 0.79±0.02 | 26.30±1.26 | 44.80±0.43 | 83.69±1.96 | 3.26±0.27 | 74.93±15.95 | 51.72±19.80 | 0.51±0.07 |
| *Microlepia platyphylla* | 1.28±0.06 | 14.71±0.76 | 52.99±0.55 | 79.05±0.92 | 3.58±0.15 | 111.66±6.41 | 77.93±15.60 | 1.03±0.07 |
| *Coniogramme fraxinea* | 1.16±0.03 | 59.03±1.64 | 47.30±0.54 | 72.88±2.32 | 4.11±0.17 | 87.97±13.55 | 148.36±15.30 | 1.08±0.09 |
| *Pteris esquirolii Christ* | 1.19±0.04 | 72.36±1.96 | 38.40±0.33 | 68.96±1.31 | 3.06±0.19 | 71.19±6.61 | 43.90±16.70 | 0.59±0.07 |
| *Gymnosphaera gigantea* | 1.36±0.03 | 200.99±5.12 | 30.92±0.39 | 75.89±0.96 | 3.76±0.17 | 83.16±3.97 | 58.39±7.30 | 0.62±0.03 |
| *Christensenia assamica* | 1.20±0.04 | 136.89±3.40 | 37.67±0.38 | 88.15±1.05 | 2.95±0.13 | 86.55±8.87 | 93.49±8.53 | 2.05±0.01 |
| *Angiopteris hokouensis* | 1.33±0.06 | 72.78±1.69 | 44.46±0.36 | 83.73±0.11 | 5.53±0.22 | 132.15±5.47 | 134.38±14.30 | 1.19±0.04 |
| *Angiopteris yunnanensis* | 1.44±0.02 | 136.46±3.19 | 38.22±0.46 | 85.09±1.19 | 4.32±0.27 | 104.20±11.13 | 91.52±14.70 | 0.89±0.04 |
| *Selaginella superha* | 0.66±0.02 | 11.69±0.42 | 25.11±0.29 | 79.71±0.74 | 2.21±0.12 | 77.41±2.23 | 16.82±10.50 | 1.52±0.07 |

See Table 1 for trait units. D_vein_, vein density; SD, stomatal density; SL, stomatal length; LWC, leaf water content; A_max_, area-based maximum photosynthetic rate; A_mass_, mass-based maximum photosynthetic rate; g_s_, stomatal conductance; T_r_, transpiration rate.

**Table S4. Pairwise cross-species and phylogenetically independent contrast (PIC) correlations between the 13 leaf functional traits for 30 fern species.**

|  | LMA | LWC | CT | UET | LET | LT | MT | SD | SL | A_max_ | g_s_ | T_r_ | D_vein_ |
| --- | --- | --- | --- | --- | --- | --- | --- | --- | --- | --- | --- | --- | --- |
| LMA |  | 0.013 | 0.226 | 0.245 | 0.345 | 0.655^***^ | 0.659^***^ | 0.193 | 0.297 | 0.238 | 0.320 | 0.358 | 0.207 |
| LWC | 0.142 |  | 0.397^*^ | 0.280 | 0.458^*^ | 0.591^***^ | 0.572^***^ | 0.252 | 0.285 | 0.022 | 0.034 | 0.177 | 0.102 |
| CT | 0.228 | 0.292 |  | 0.018 | 0.386^*^ | 0.350 | 0.344 | 0.056 | 0.027 | 0.075 | 0.058 | 0.213 | 0.202 |
| UET | 0.168 | 0.407^*^ | 0.112 |  | 0.573^***^ | 0.055 | 0.130 | 0.335 | 0.308 | 0.279 | 0.018 | 0.295 | 0.165 |
| LET | 0.201 | 0.451^*^ | 0.386^*^ | 0.688^***^ |  | 0.538^**^ | 0.460^*^ | 0.216 | 0.323 | 0.297 | 0.185 | 0.304 | 0.088 |
| LT | 0.464^*^ | 0.606^***^ | 0.404^*^ | 0.044 | 0.455^*^ |  | 0.995^***^ | 0.309 | 0.454^*^ | 0.157 | 0.276 | 0.231 | 0.060 |
| MT | 0.467^*^ | 0.581^***^ | 0.493^*^ | 0.038 | 0.364 | 0.994^***^ |  | 0.294 | 0.427^*^ | 0.119 | 0.256 | 0.268 | 0.048 |
| SD | 0.051 | 0.301 | 0.077 | 0.186 | 0.036 | 0.121 | 0.126 |  | –0.731^***^ | 0.450^*^ | 0.437^*^ | 0.441^*^ | 0.531^**^ |
| SL | 0.058 | 0.380^*^ | 0.016 | 0.195 | 0.135 | 0.293 | 0.274 | –0.502^**^ |  | 0.265 | 0.264 | 0.253 | 0.220 |
| A_max_ | 0.203 | 0.042 | 0.043 | 0.212 | 0.331 | 0.048 | 0.003 | 0.052 | 0.095 |  | 0.634^***^ | 0.418^*^ | 0.522^**^ |
| g_s_ | 0.091 | 0.094 | 0.231 | 0.140 | 0.007 | 0.041 | 0.024 | 0.140 | 0.202 | 0.523^**^ |  | 0.716^***^ | 0.293 |
| T_r_ | 0.220 | 0.175 | 0.264 | 0.543^***^ | 0.563^**^ | 0.080 | 0.142 | 0.177 | 0.162 | 0.095 | 0.621^***^ |  | 0.249 |
| D_vein_ | 0.234 | 0.049 | 0.310 | 0.286 | 0.111 | 0.048 | 0.066 | 0.479^**^ | 0.045 | 0.371^*^ | 0.203 | 0.109 |  |

Correlation data are given for species-based analyses above the diagonal and for PIC analyses below the diagonal. The sign of the significance for each correlation is indicated as: ^*^, *p* < 0.05; ^**^, *p* < 0.01; ^***^, *p* < 0.001.

LMA, leaf mass per unit area; LWC, leaf water content; CT, cuticle thickness; UET, upper epidermal thickness; LET, lower epidermal thickness; LT, leaf thickness; MT, mesophyll thickness; SD, stomatal density; SL, stomatal length; A_max_, area-based maximum photosynthetic rate; g_s_, stomatal conductance; T_r_, transpiration rate; D_vein_, vein density.
